# The diagnostic predictive value of neutrophil-to-lymphocyte ratio in thyroid cancer adjusted for tumor size

**DOI:** 10.1371/journal.pone.0251446

**Published:** 2021-05-11

**Authors:** Taek Yoon Cheong, Sang Duk Hong, Keun-Woo Jung, Yoon Kyoung So

**Affiliations:** 1 Department of Otorhinolaryngology–Head & Neck Surgery, Inje University College of Medicine, Ilsan Paik Hospital, Goyang-Si, Korea; 2 Department of Otorhinolaryngology-Head and Neck Surgery, Samsung Medical Center, Sungkyunkwan University School of Medicine, Seoul, Korea; Universidade do Porto Faculdade de Medicina, PORTUGAL

## Abstract

The role of systemic inflammation has not been clearly defined in thyroid cancers. There have been conflicting reports on whether systemic inflammatory markers have predictive value for thyroid cancers. We aimed to evaluate the association between systemic inflammatory markers and clinicopathological factors in thyroid cancers and to assess their predictive value for thyroid cancers in detail. Five hundred thirty-one patients who underwent surgery for thyroid nodules were included. The patient population consisted of 99 individuals (18.6%) with benign thyroid nodules and 432 individuals (81.4%) with thyroid cancers. In 432 patients with thyroid cancers, neutrophil-to-lymphocyte ratio (NLR) was significantly higher in the cases with tumors greater than 2 cm than in those with tumors less than 2 cm. (*p* = 0.027). NLR and platelet-to-lymphocyte ratio (PLR) were significantly higher in cases with lateral lymph node metastasis (LNM) than in those without LNM (*p* = 0.007 and 0.090, respectively). The nodule size was significantly higher in benign thyroid nodules than in thyroid cancers (*p* < 0.001). When the cases were stratified by tumor size, NLR was a significant predictor of thyroid cancers in cases with nodules greater than 2 cm (Exp(B) = 1.85, 95% CI = 1.15–2.97, *p* = 0.011), but not in those with nodules less than 2 cm. In thyroid cancers, preoperative NLR was associated with pathological prognosticators such as tumor size and lateral lymph node metastasis. When the size difference between thyroid cancers and benign thyroid nodules was adjusted, NLR could be a significant predictor of thyroid cancers.

## Introduction

Systemic inflammatory markers are known to have prognostic value for various cancers including head and neck, hepatocellular, urinary, and biliary cancers [1–5]. However, their roles have not been clearly defined in thyroid cancers. First, their prognostic implication is still unclear and is difficult to evaluate due to this cancer’s extremely low recurrence rate and the high survival rate of thyroid cancers, although systemic inflammatory markers have been reported as prognostic markers in papillary thyroid cancers by some authors [6, 7]. Next, there is a lack of investigation of their association with the adverse clinicopathological features of thyroid cancers, such as extrathyroidal extension and lymph node metastasis [8, 9]. Last, there have been conflicting reports on whether the systemic inflammatory markers are predictive of thyroid cancer [10, 11]. The considerable heterogeneity in the differentiation of the included thyroid cancers in the previous studies may be the cause of these inconsistent and ambiguous results. In this study, we aimed to investigate the association between systemic inflammatory markers and clinicopathological factors in thyroid cancers and to assess their diagnostic predictive value for thyroid cancers.

## Materials and methods

This study was approved by the Institutional Review Board of Ilsan Paik Hospital (File No. 2020-07-046) and was conducted in accordance with the Helsinki Declaration. Informed consents were waived because of the retrospective design of this study. Five hundred seventy-three patients who had thyroidectomy from January 2009 to September 2019 were initially reviewed. Patients’ medical records were reviewed, and the data was analyzed from July 2020 to December 2020. Patients who had surgery for thyrotoxicosis were not included. Also, we excluded cases in which the surgical pathology was just an inflammatory or autoimmune disease such as Graves’ disease or Hashimoto’s thyroiditis. Five hundred thirty-one patients who underwent surgery for thyroid nodules were finally included in this retrospective study. Thyroid lobectomy was performed for benign thyroid mass. For suspected malignancy, the extent of surgery (lobectomy or total thyroidectomy and lymph node dissection or not) was determined by extent of disease.

Data from blood tests that were checked within 4 weeks before surgery were obtained. White blood cell (WBC) count, neutrophil ratio, lymphocyte ratio and platelet count were obtained. Reference ranges for the results were 4.00–10.00/mL for WBC count, 43.0–70.0% for neutrophil ratio, 20.0–44.0% for lymphocyte ratio and 150-400/mL for platelet count. All blood tests were analyzed by XE-2100 differential analyzer (Sysmex, Japan). Quality assurance of test result was proved by Korean Association of External Quality Assessment Service. Neutrophil-to-lymphocyte ratio (NLR) and platelet-to-lymphocyte ratio (PLR) were calculated by dividing neutrophil count and platelet count by lymphocyte count, respectively. Demographic and clinicopathological characteristics were obtained from patients’ medical records; age, sex, surgical pathology, the size of the largest nodule, and presence of coexisting chronic lymphocytic thyroiditis. In cases of thyroid cancers, information on tumor grade and extent were also obtained; extrathyroidal extension (ETE), central lymph node metastasis (CNM), lateral lymph node metastasis (LNM), and multifocality. Pathologically, papillary thyroid carcinomas (PTC) and follicular thyroid carcinomas (FTCs) are classified as differentiated thyroid cancers (DTCs).

### Statistical analysis

The Wilcoxon rank-sum test was used to compare continuous variables. A normality test on continuous variables was performed using the Kolmogorov-Smirnova test. Correlations between continuous variables were analyzed using Spearman bivariate correlation analysis. Binary logistic regression test was used to assess the predictive value of NLR and PLR for thyroid malignancy. SPSS software for Windows, version 17.0 (SPSS Inc., Chicago, IL) and R 3.6.1 for Windows were used for statistical analyses. All tests were 2-sided and p < 0.05 was considered statistically significant.

## Results

### Patients’ characteristics

The mean age of all patients was 50.5 years. Of 531 patients, 404 (76.1%) were female and 127 (23.9%) were male. Pathologically, benign thyroid nodules accounted for 18.6% (n = 99) and thyroid cancers for 81.4% (n = 432). Mean size of nodules was 1.5 cm. There were 398 cases with nodules less than 2cm and 133 with nodules of 2cm or more. Of 398 cases with mass less than 2cm, 377 (94.7%) were malignant and 21 (5.3%) were benign. Of 133 cases with nodules of 2cm or more, 55 (41.4%) were malignant and 78 (58.6%) were benign. Of 432 cases with thyroid cancers, 421 (97.5%) were PTCs. There were 5 FTCs, 3 anaplastic thyroid carcinomas (ATCs), 1 medullary thyroid carcinoma (MTC), and 2 carcinomas showing thymus-like differentiation (CASTLEs). ETE, CNM, and LNM were present in 231 (53.5%), 134 (31.0%), and 29 (6.7%) of 432 patients with thyroid cancers, respectively (Table 1).

**Table 1:** Patients’ characteristics.

Variables		Values
No. of patients		531
Age, yr	mean ± SD	50.5 ± 13.6
	< 45	161 (30.3)
	≥ 45	370 (69.7)
Sex	Male (%)	127 (23.9)
	Female (%)	404 (76.1)
Pathology	Benign	99 (18.6)
	Malignant	432 (81.4)
	PTC	421
	FTC	5
	others	6
Size of mass, cm	mean ± SD	1.5 ± 1.3
	< 2 (%)	398 (75.0)
	≥ 2 (%)	133 (25.0)
Thyroiditis	Absent (%)	476 (89.6)
	Present (%)	55 (10.4)
ETE**	Absent (%)	201 (46.5)
	Present (%)	231 (53.5)
CNM**	Absent (%)	298 (69.0)
	Present (%)	134 (31.0)
LNM**	Absent (%)	403 (93.3)
	Present (%)	29 (6.7)

SD, standard deviation; ETE, extrathyroidal extension; CNM, central nodal metastasis; LNM lateral nodal metastasis.

* The diameter of a largest nodule with representative pathology.

** Values are presented for cases with thyroid cancers.

### Association between systemic inflammatory markers and clinicopathological characteristics in thyroid cancers

**Fig 1:**
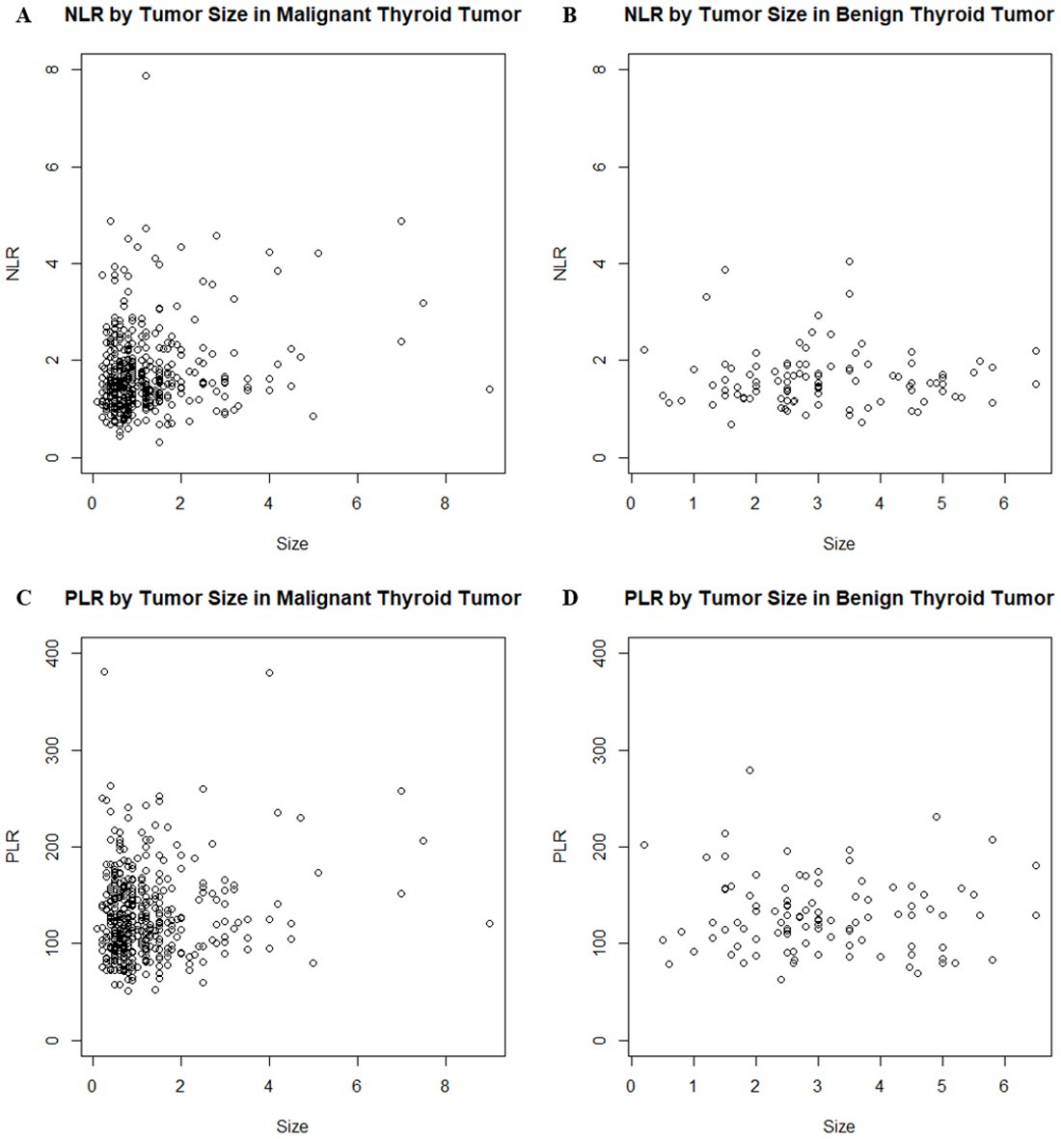
Correlation between tumor size and systemic inflammatory markers in benign thyroid nodules and thyroid cancers. In thyroid cancers, tumor size weakly correlated with NLR (*rho* = 0.097, *p* = 0.044) (A). There was no significant correlation between PLR and tumor size in thyroid cancers (*rho* = 0.045, *p* = 0.347) (C). In benign thyroid nodules, the nodule size did not have significant correlation with NLR or PLR (B, D). NLR, neutrophil-to-lymphocyte ratio.

Clinicopathological characteristics for 432 patients with thyroid cancers were analyzed in relation to NLR and PLR (Table 2). NLR was significantly higher in patients with tumors greater than 2 cm than in those with tumors less than 2 cm. (*p* = 0.027). Tumor size weakly correlated with NLR (*rho* = 0.097, *p* = 0.044, Fig 1A). There was no statistically significant difference in PLR between the two tumor size groups (*p* = 0.239, Table 2), and no significant correlation between PLR and tumor size (*rho* = 0. 045, *p* = 0.347, Fig 1C). Among adverse pathological features, only LNM was significantly associated with NLR (*p* = 0.007). Although PLR were slightly higher in cases with LNM than in those without LNM, there was no statistical significance (*p* = 0.090). CNM, ETE, coexisting thyroiditis, and tumor multifocality were not significantly associated with NLR or PLR. A significant association between sex and PLR was observed (*p* < 0.001).

**Table 2:** Association between systemic inflammatory markers and clinicopathological characteristics in thyroid cancers.

		NLR*	*p* value	PLR*	*p* value
Age	<45yrs	1.58 (1.20~2.02)	0.259	120.87 (103.37~154.12)	0.560
	≥45yrs	1.51 (1.21~1.95)		121.34 (96.61~148.47)	
Sex	Female	1.53 (1.20~2.00)	0.931	124.58 (102.66~154.08)	**<0.001**
	Male	1.56 (1.27~1.93)		105.97 (88.98~138.52)	
Tumor size	<2cm	1.52 (1.18~1.96)	**0.027**	120.52 (97.94~147.37)	0.239
	≥2cm	1.61 (1.40~2.24)		124.62 (98.99~159.49)	
ETE	Absent	1.53 (1.15~2.01)	0.264	119.44 (97.52~150.75)	0.568
	Present	1.54 (1.29~1.96)		121.82 (98.42~148.90)	
CNM	Absent	1.53 (1.17~1.97)	0.197	119.98 (97.83~148.67)	0.217
	Present	1.56 (1.30~2.04)		124.52 (100.20~153.62)	
LNM	Absent	1.53 (1.20~1.95)	**0.007**	120.87 (97.52~147.39)	0.090
	Present	1.67 (1.47~3.58)		126.28 (105.26~172.91)	
Multifocality	Absent	1.53 (1.20~1.97)	0.511	119.07 (97.83~149.15)	0.457
	Present	1.55 (1.23~2.10)		126.13 (98.85~149.17)	
Thyroiditis	Absent	1.54 (1.21~2.01)	0.131	121.51 (98.02~149.12)	0.511
	Present	1.48 (1.20~1.68)		120.71 (94.54~154.13)	

NLR, neutrophil-to-lymphocyte ratio; PLR, platelet-to-lymphocyte ratio; ETE, extrathyroidal extension; CNM, central nodal metastasis; LNM lateral nodal metastasis.

* Values are presented as median (1^st^ quartile ~3^rd^ quartile).

### Systemic inflammatory markers and the prediction of thyroid cancers

While NLR correlated with tumor size in thyroid cancers (*rho* = 0.097, *p* = 0.044, Fig 1A), size of benign thyroid nodules did not correlate with NLR (*rho* = 0.057, *p* = 0.573, Fig 1B).

**Fig 2:**
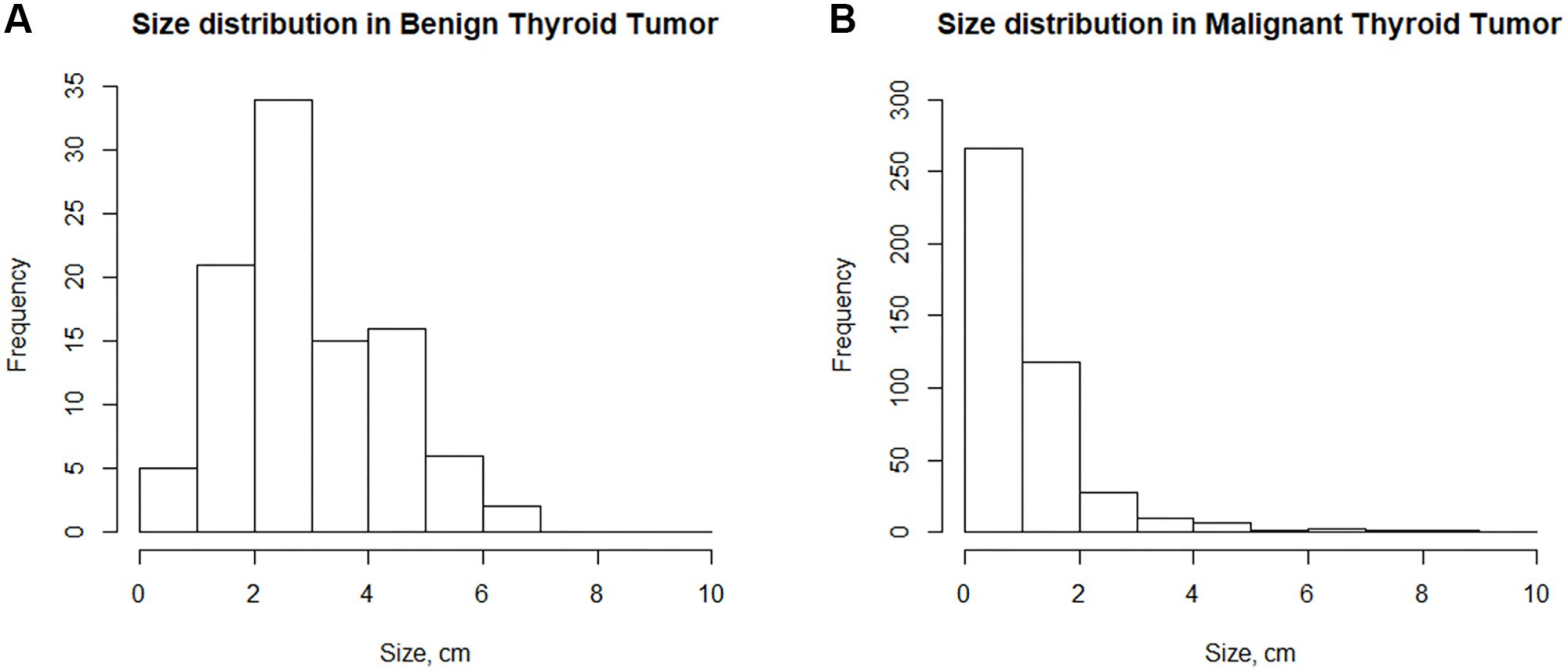
Size distribution of benign thyroid nodules and thyroid cancers. The median nodule size was 0.8 cm in thyroid cancers (B) and 2.8 cm in benign thyroid nodules (A). Nodule size was significantly larger in benign thyroid nodules than in thyroid cancers (*p* < 0.001, Wilcoxon rank-sum test).

The distribution of nodule size was largely different between thyroid cancers and benign thyroid nodules. Nodule size was not normally distributed in both groups (*p* < 0.001 for both groups, Kolmogorov-Smirnova test). The median nodule size was 0.8 cm in thyroid cancers and 2.8 cm in benign thyroid nodules (Fig 2). Nodule size was significantly larger in benign thyroid nodules than in thyroid cancers (*p* < 0.001, Wilcoxon rank-sum test). Factors other than nodule size were not significantly different between the two groups. Female predominance was similar in both groups (76.4% in the thyroid cancer group and 74.7% in the benign thyroid nodule group, *p* = 0.730). Patient median age was 49 in the thyroid cancer group and 51 in the benign thyroid nodule group (*p* = 0.361). Thyroiditis was present in 11.1% of the thyroid cancer group and in 7.1% of the benign thyroid nodule group (*p* = 0.234).

NLR and PLR were not statistically significant predictors for thyroid cancers (Table 3) in the overall patients. The median NLR was 1.54 in the thyroid cancer group and 1.53 in the benign thyroid nodule group (*p* = 0.838). The median PLR was 121.20 in the thyroid cancer group and 124.96 in the benign thyroid nodule group (*p* = 0.466). Considering the difference in nodule size between the two groups, the cases were stratified by size group (Table 3). NLR was a significant predictor of thyroid cancers in cases with tumors greater than 2 cm (median NLR 1.61 in the thyroid cancer group and 1.55 in the benign thyroid nodule group; Exp(B) = 1.85, 95% CI = 1.15–2.97, *p* = 0.011), but not in those with tumor less than 2 cm. The same analysis was conducted for only DTCs after excluding ATCs, MTCs, and CASTLEs. Likewise, NLR was a significant predictor of DTCs in cases with tumors greater than 2 cm (Exp(B) = 1.68, 95% CI = 1.02–2.78, *p* = 0.043), but not in those <2 cm. PLR was not a significant predictor of thyroid cancers or DTCs.

**Table 3:** Prediction of thyroid cancers with systemic inflammatory markers.

	Prediction of thyroid cancers			Prediction of DTCs		
	Exp(B)	95% CI	*p* value	Exp(B)	95% CI	*p* value
*In all cases (N = 531*, *malignancy = 432*, *DTC = 426)*						
NLR	1.161	0.858–1.572	0.333	1.138	0.838–1.546	0.408
PLR	0.999	0.994–1.004	0.766	0.999	0.994–1.004	0.702
*In cases ≥ 2cm (N = 133*, *malignancy = 55*, *DTC = 50)*						
NLR	1.848	1.153–2.967	**0.011**	1.681	1.017–2.778	**0.043**
PLR	1.006	0.998–1.014	0.136	1.005	0.997–1.013	0.233
*In cases < 2cm (N = 398*, *malignancy = 377*, *DTC = 376)*						
NLR	1.087	0.593–1.992	0.787	1.091	0.595–2.000	0.780
PLR	0.994	0.985–1.003	0.176	0.994	0.985–1.003	0.178

DTC, differentiated thyroid carcinoma; NLR, neutrophil-to-lymphocyte ratio; PLR, platelet-to-lymphocyte ratio; CI, confidential interval.

## Discussion

Systemic inflammatory responses can suppress the anti-tumor activity of immune cells, such as activated T cells and natural killer cells [12, 13]. Although underlying mechanisms are not clearly defined, surrogate markers for systemic inflammation, such as neutrophil, lymphocyte, and platelet counts, either alone or expressed as their ratios, has been reported to be associated with prognosis for various cancers [14–21]. Among those markers, NLR is known to have superior prognostic value compared to the other individual leukocyte counts [4, 22]. NLR has been reported to be a significant prognosticator for head and neck cancers in recent studies [4, 5, 22]. However, the significance of those systemic inflammatory markers including NLR is still unclear in thyroid cancers. Several studies investigated the association between systemic inflammatory markers and prognosis for thyroid cancers. Jung et. al reported that 5-year disease-free survival (DFS) rate was significantly worse in stages III and IV PTC patients with NLR ≥ 1.5 compared to those with NLR < 1.5 (94.1 vs. 99.3%, *p* = 0.013) [6]. Yokota et. al also reported that low lymphocyte-to-monocyte ratio (LMR) was associated with recurrence, especially in patients with advanced PTC [7]. However, Lang et. al showed that there was no significant association between a high NLR and poor DFS in cN0 PTC [23]. Compared to other head and neck cancers, studies on the prognosis of thyroid cancers are difficult due to their extremely good prognosis. The 10-year relapse-free survival for scan-negative patients with PTC after thyroidectomy was reported to be about 90% [24]. The 10-year overall survival for PTC was reported to reach 97% [25]. Considering that DTCs account for the majority of thyroid cancers cases and that early-stage PTCs or papillary thyroid microcarcinomas (PTMCs) account for the majority of the DTC cases, it is possible to guess why prognostic studies in thyroid cancer are difficult. Twenty-year recurrence rate was only 6% in PTMC [26]. Although the prognostic value of systemic inflammatory markers for advanced thyroid cancer has been reported in some studies [6, 27], it is difficult to conduct large-scale studies with relevant clinical implications due to the low incidence of advanced thyroid cancers. It might be more practical to study the association between systemic inflammatory markers and clinicopathological prognosticators of thyroid cancer, rather than the disease-specific events themselves such as death or recurrence. Manatakis et al. reported that NLR was significantly associated with ETE and multifocal tumors, as well as lymph node metastasis. Kim et al. also reported that PLR was positively associated with larger tumor size and LNM. There have not been enough studies on this issue. In this study, NLR and PLR were significantly associated with tumor size and LMN in the cases with thyroid cancer. Although statistical analysis was not performed due to the small number of cases, NLR and PLR were noticeably high in ATCs compared with DTCs. Mean NLR (±SD) was 4.09 ± 0.49 in ATCs, 1.70 ± 0.39 in PTCs, and 1.79 ± 0.36 in FTCs. Similarly, mean PLR (±SD) was 212.23 ± 24. 63 in ATCs, 127.53 ± 2.10 in PTCs, and 142.98 ± 30.29 in FTCs.

In fact, studies on the role of systemic inflammatory markers in thyroid cancer inevitably focus on their predictive values for the diagnosis of thyroid cancers. There is still controversy as to whether systemic inflammatory markers can differentiate between thyroid cancer and benign thyroid nodules. A recent meta-analysis that combined the outcomes of 6,283 patients with thyroid nodules (4,617 DTCs and 1,666 benign thyroid nodules) from 6 individual studies showed that the pretreatment NLR values were not significantly different between patients with DTC and those with benign nodules [10]. However, there was significant heterogeneity in the patient population among the included individual studies. Depending on whether ATCs or undifferentiated carcinomas are included, NLR or PLR can vary greatly and their predictive values for thyroid cancers can be different. More importantly, the previous studies on the predictive values of systemic inflammatory markers did not take into account the size distribution of thyroid nodules. As shown in this study and reported previously, tumor size in thyroid cancers can affect systemic inflammation. If PTMCs account for the majority of included thyroid cancer cases, the difference in NLR or PLR between the thyroid cancers and benign thyroid nodules cannot reach statistical significance. Since benign thyroid nodules are resected because of their large size in many cases, tumor size in the benign nodule group is inevitably larger than that in the thyroid cancer group. The size difference can be much larger if small PTMCs account for the majority of included thyroid cancer cases. The difference in NLR or PLR between the thyroid cancers and benign thyroid nodule groups could not reach statistical significance for this reason. In this study, the median nodule size was 0.8 cm for thyroid cancers and 2.8 cm for benign thyroid nodules (*p* < 0.001). Although NLR was not a significant predictor of thyroid cancers in overall patients, statistical significance was found in patients with tumors greater than 2 cm (Table 3). Adjunctively, for tumors less than 2cm, the difference in the number of samples in the two groups should be noted (21 in benign thyroid nodule group *vs*. 378 in thyroid cancer group). The noticeable difference of sample size could limit the statistical significance for tumors less than 2cm.

There are some limitations in this study. The limitations are mainly related to its retrospective design. First, the intrinsic variability of the blood test values should be addressed. Compared to individual blood test values such as neutrophil count, NLR has been reported to have superior stability under various physiological conditions and during in vitro handling of blood samples [22, 28]. Nevertheless, NLR and PLR also inevitably have variability. This variability can be adjusted to some extent by a large-volume study or repeated tests within an individual patient. Second, selection bias regarding benign nodules is still possible in this study. Unlike thyroid cancers which are operated in most cases, benign thyroid nodules are resected in limited cases. Operated cases are mainly those with huge nodules or with the potential risk of malignancy. On the contrary, cases with tumor greater than 2 cm represent the very minority of the cohort in thyroid cancer group. Although tumor size difference was adjusted by stratification, there may be a selection bias regarding benign nodules. To overcome these limitations and to have more clear results, a large-volume, prospective, controlled study is required.

## Conclusion

In thyroid cancers, preoperative NLR is associated with pathological prognosticators such as tumor size and LNM. If the size difference between thyroid cancers and benign thyroid nodules is adjusted, NLR can be a significant predictor of thyroid cancer. A prospective controlled study is required to confirm these results.

## Supporting information

S1 File (XLSX)
